# A Physiological Memoir upon the Brain

**Published:** 1828-11

**Authors:** 


### REVIEWS.

---

Art. II.—*A physiological memoir upon the Brain.* By M. Majendie. *Read in a public sitting of the Royal Academy of Sciences, on the* 16*th of June*, 1828. From the French. By Joseph Gardener, M. D.

Some old writer has compared Anatomy to Geography:—Geology would perhaps have afforded a better object of comparison, but Geology was then unknown as a science. The age of *geographical* discovery is gone by, and so it is, we opine, with the brilliant era of *anatomical* discovery. The spirit of man remains, however, the same; and we still see him as intent as ever, in the *search* after new organs in the living body, and new lands in the vast solitudes of the ocean. In both cases, at the present day, his utmost efforts must, generally, eventuate in disappointment, for the best possible reason—that the great world has been explored by preceding geographers, and the microcosm or little world of man, by preceding anatomists. In both, the *ratio* of success is a diminishing ratio; and must at last evanish altogether. Just in proportion, however, to the difficulty of finding any thing new, is the importance of a discovery enhanced, in the estimation of those who make it; and, what is of greater moment to be borne in mind, the credulity of professional discoverers exalted. Hence we should turn, not a deaf ear, but an ear of unbelief, or at least of doubt, to every rumour of new discovery, not backed by the testimony of a 'cloud of witnesses.' When all was *unexplored*, but little evidence could be required to secure our belief in the existence of all that was said to have been seen; but since nearly all *has been* explored, a philosophical incredulity, should be cherished in reference to every alledged discovery.

In most parts of the human body the search after novelties is given up, but the central portions of the nervous system, still present, or seem to present, some tracts of *terra incognita,* and to these has the adventurous spirit of anatomical research, of late, chiefly directed its movements. Of those who have obeyed, and are still obedient to this spirit, none are more distinguished for industry, boldness, and perseverence, than the ingenious physiologist whose latest memoir we are about to introduce to the notice of our readers. According to this report, he has discovered within the cranium and spinal column of the living animal, in a state of health, a fluid anatomical element, distinct from the blood, and scarcely less necessary than that liquid, to the manifestation of the phenomena of animal life. It exists, according to his declaration, not only in the ventricles, but also *around* the brain and spinal marrow and origins of all the nerves, passing with facility from the centre to the circumference, *et vice versa.* This liquid he denominates spino-cephalic, or cephalo-rachidian. Its quantity, in an adult of ordinary stature and in good health, is estimated at about three ounces; in women, *caeteris paribus,* something more. In old men the proportion is still greater, in some instances amounting to six or seven ounces; but under such circumstances the mental and corporeal energies are enfeebled. The layer which it forms around the brain and spinal marrow varies in thickness in different places, being in the neck four or five lines, in the lumbar region more than an inch, and around the brain from one to two lines.

Majendie asserts, that the volume of the brain is not invariably the same, as has generally been supposed, but that it varies like the volume of the other organs of the body. That when the body becomes emaciated, the brain undergoes a like diminution, and as the emaciated organs regain their former size, it also recovers what it lost. But no vacuum occurs in the cavity of the skull under any circumstances; hence, the first office of this liquid is to fill up the space produced by the diminished size of the brain, as often as that occurrence takes place. When the viscera of the thorax and abdomen diminish in volume, their parieties being flexible and pressed upon by the atmosphere, follow the retreat of those organs, and a vaccuum is thus prevented. But the cranium being inflexible, does not follow the brain when it decreases; and hence the first use assigned for the cephalo-spinal liquid.

Its second office is to control the powers of life. Experiments were instituted upon animals, to test this point. A fierce fox was taken, and a small puncture was made in the nape of the neck, and the cephalo-spinal fluid was quickly evacuated; the animal became suddenly calm, and lay upon the ground without the least movement. After the expiration of 36 hours, it began to move, and attempted to bite and to make its escape. A new puncture was then made, and the cephalo-spinal liquid, which had been speedily reproduced, was allowed to escape; the animal then exhibited the same symptoms as in the former experiment.

In the disease of infants, where a sac filled with water occurs at the inferior extremity of the spine, Majendie thinks, this sac is a hernia, containing the cephalo-spinal liquid. When the sac is punctured, death is the immediate consequence; because the puncture remaining open, the liquid escapes, and ceases to protect the spinal marrow and brain.

Thus, says he, not only in man, but in animals, this liquid preserves the integrity of the nervous functions, and is necessary for the continuance of life. It is thought likewise, that it not only exercises an influence over the functions of the nervous system, by its contact with the brain and spinal marrow, but by its chemical properties, and its temperature.

It is the opinion of practical anatomists, that the cavities of the brain, or its ventricles, contain little or no serum in their healthy state. Nevertheless, when we open the brain, we find its ventricles almost always filled with a limpid fluid. Majendie thinks it is the same kind of fluid that is found at the surface of the brain and spinal marrow, and believes that it naturally exists in all these places. It was necessary for confirming the identity of these fluids, to discover a communication between the exterior of the organ and its internal cavities. This, Majendie says, he has accomplished, and that there exists an opening of 3 or 4 lines in diameter, completely concealed by a lobe of the cerebellum, and forming a true entrance into the ventricles. Hence it becomes mechanically necessary, that the cephalo-spinal liquid should enter into the cavities of the brain, and fill them, in consequence of their communicating with one another. From these circumstances, it is inferred, that the great valve of the brain, as it is called by anatomists, does in reality perform, to a certain extent, the office of a *valve*. The aqueduct of sylvius being overhung by the valve, and its size somewhat regulated by it, conveys this liquid from the 4th to the 3d ventricle, and the infundibulum conveys it to the pituitary gland.

The cephalo-spinal liquid is thought never to be in a state of repose, but continually agitated by the *flux* and *reflux* of the brain, which takes place from the influence of respiration. Thus, when air is received into the thorax, a portion of this liquid flows from the cerebral cavities into the spinal canal; and when it is driven from the lungs, the liquid re-enters these cavities, by traversing the passages above mentioned. This flux or reflux is probably owing, he thinks, to the alternate swelling and depression of the veins; the circulation of whose blood is under the influence of respiration.

The office assigned the pineal gland is that of a *tampon*, for the purpose of opening and closing the aqueduct of the brain. This gland is situated above the anterior opening of the aqueduct. Two voluminous veins are firmly placed upon the gland. These veins vary in size, being sometimes very much swelled, at others nearly empty. Hence, at the moment the veins are swelled, they must press down the pineal gland and close the opening of the aqueduct. And as one of the constant effects of violent exercise, rage, crying, &c. is to swell the veins of the head, it results, that the entrance of the cephalo-spinal liquid into the cavities of the brain is intercepted or modified, according to circumstances.

Lastly, Majendie proceeds to consider, what influence the cephalo-spinal liquid exercises over the intellectual faculties. To determine this point, it became necessary to ascertain the quantity of the cephalo-spinal liquid, 1st in the sane, 2d in the imbecile, 3d in idiots. Majendie had an ample opportuntiy of determining the quantity in the latter, at the *Salpetriere*, where are collected together a great number of fools and idiots. In idiots the liquid is found to occupy the surface of the brain and spinal marrow, under the form of a thick layer, and it also distends the ventricles, and displaces all the organs connected with them, especially the pineal gland. In these cases, the quantity amounted to 6 or 7 ounces, and the same circumstances obtain, in cases of old persons, who suffer from mental derangement.

In the insane and the imbecile from insanity, the liquid does not accumulate upon the surface of the brain, but in its cavities. In all cases of derangement, whether furious mania, or melancholia, the ventricles are always much distended. Persons endowed with reason have less than one ounce in the ventricles at the time of death.

From these general results the fact is inferred, that the development of the mind is in an inverse ratio to the quantity of the cephalo-spinal liquid.

By this discovery of an exterior cephalic fluid in the normal state of the body, our author conceives that the whole doctrine of *organology* is washed away, and of course the practical phrenologist is left without any foundation to stand upon. Such, briefly, are the principal contents of the memoir before us, a fuller notice of which seems unnecessary.

The intelligent reader will perceive that it sets forth two discoveries, 1st, a fluid surrounding the brain of the living and healthy animal, exterior to the arachnoid membrane; and 2dly, an aperture or channel through which this fluid circulates into and from the ventricles. Now we are not prepared to reject the reality of the latter discovery, as an aperture a few lines in diameter in a pulpy mass, like that of the brain, may have escaped previous observation; but that so many distinguished and prying anatomists as have dissected that organ, should not have recognized the existence of the exterior fluid in the healthy state, seems at least an improbability. If it should be said that at or after death this fluid retreats to the ventricles, some adequate cause ought certainly to be shown for such a movement; and until this is done, a belief in the assertion must be gratuitous. That Majendie has repeatedly found a serous fluid surrounding the brain, no one can doubt.

Others, as we have already said, have met with the same thing. The question is, whether it is a component part of the body in health? For ourselves, we are compelled to say, that the facts contained in his memoir do not incline us to adopt the affirmative of this question. He has seen it most abundant in those whose cerebral functions were disordered in life. May it not then, in all cases, be the offspring of a morbid action in the encephalic membranes? If it were always present, how could it have escaped the notice of the surgeons who have so often witnessed læsions of the dura mater, in persons, who at the time of the accidents which befell them were in perfect health?

As to the objection which his discovery, supposing it to be rëal, presents to the very doubtful science of phrenology, we regard it as of no weight. If the fluid is spread over the surface of the whole brain, the relative development of its different parts or convolutions, might still be the same as if its surface came directly in contact with the dura mater. If the speculations in *organology*, therefore, laboured under no greater difficulties, than that which our author has started, they would rest on a firmer basis than that on which they seem to us to repose.

That the brain is liable to a degree of partial or general atrophy, is undoubtedly a fact; but this is the effect of disease, and the same morbid action in all probability gives rise to the serous accumulations, which our author attributes to a *vis conservatrix*, when he speaks of an increased secretion of the cephalo-rachidian fluid as designed to compensate for the absorption of the encephalic substance, and to preserve a cranial *plenum*. That the brain is subject to an increase and decrease of magnitude, whenever, from any cause, the body at large is brought to vary in its magnitude, is a position which practical anatomy does not sustain. The other organs derive a great part of their bulk from the cellular membrane which binds together the vessels and fibres which constitute their essential component parts; and the absorption of that membrane may go on till these organs suffer a great diminution of size; but in the brain, that tissue is not present; and whatever absorption takes place, must be of the pulpy matter itself; and hence nature seems to have provided, that under ordinary causes of emaciation, the cerebral absorbents should not be called into action; in this consists the preservative principle of a cranial *plenum*, and not in the increased secretion of a spino-cephalic fluid.

Before closing this notice of the memoir of Majendie, we consider it due to his high standing, in the physiological community, to furnish our readers with an extract; and shall select those paragraphs which set forth his views of the influence of the spino-cephalic fluid upon our intellectual functions.

"The liquid which fills the cavities of the brain is never in repose—it experiences a continual agitation in consequence of a kind of flux and reflux, which takes place under the influenee of respiration. Thus, at the moment when we draw the air into the thorax for the purpose of respiration, a proportion of the liquid flows from the cerebral cavities into the spinal canal—at the moment, on the contrary, when we drive the air from our lungs by expiration, the liquid re-enters these cavities by traversing the passages above mentioned, and particularly by running through the aqueduct, which thus at different times conducts the liquid in opposite directions.

The mechanical cause of the flux and reflux of the cephalo-spinal liquid is very simple—it is produced by the alternate swelling and depression of the veins, caused by the blood under the influence of respiration. This movement of the liquid may be arrested, or at least much retarded by compressing the abdomen—and this will explain the cause of belts proving injurious and even dangerous, whenever the pressure which they exercise is too considerable.

Whilst observing the movement of the liquid through the aqueduct, I believe I discovered the probable use of the pineal gland, a small body placed in the centre of the brain, and which has preserved a certain degree of celebrity since the time of Descartes.

This philosopher, who, notwithstanding the extent and vigour of his mind, yielding frequently to the propensity which we ourselves experience, in filling with our illusions the immense space which is beyond the boundary of vision and intellect, has given an hypothesis, not upon the throne of the soul, as has been remarked, but upon the situation in which it exercises its functions, and upon the throne of the imagination and of common sense, all of which he places in the pineal gland.

Voltaire, who delights in metaphysics, but loves still more to mock metaphysicians, has made a facetious parody upon the supposition of Descartes, which has been more successful than the hypothesis itself—for anatomists to the present day apply the term—reins of the soul to two nervous peduncles, which according to Voltaire, are the guides by which the pineal gland, which he compares to a coachman, directs the movements of the two hemispheres of the brain.

The functions of the pineal gland, which I propose to substitute for the hypothesis of Descartes, are very humble—but I believe them to be true, and that is a merit which, in science, ought to precede every thing. I regard the pineal gland as a tampon, for the purpose of opening and closing the aqueduct of the brain. The gland is, in fact, placed above the anterior opening of the aqueduct. Two voluminous veins are firmly placed upon the gland. These veins vary in size, being sometimes very much swelled, and at others nearly empty. It is inevitable, from the relative position of the parts that, at the moment when the veins are swelled, they must press down the pineal gland, and that this can not take place without closing more or less tightly the entrance of the aqueduct of the brain. But as one of the constant effects of violent exercise, crying, rage, and the exercise of any of the passions, is to swell considerably the veins of the head, and particularly those which press upon the pineal gland, it results, that in three different conditions, the entrance of the cephalo-spinal liquid into the ventricles must be intercepted, or at least rendered much more difficult.

The office, or to speak more correctly, one of the offices of the pineal gland, must be, therefore, an indispensable mechanical agent, for the purpose of more or less completely closing the aqueduct of the brain, and to modify, according to circumstances, the motion of the cephalo-spinal liquid, as it enters or flows from the cerebral cavities.

I come now to speak of the most important, and at the same time, the most interesting question which the study of the cephalo-spinal liquid can possibly present. What influence has this fluid upon the exercise of the intellectual faculties?

In a research, so delicate as this, and one, which presents a prospect of arriving at the most important and interesting results, the chances of committing error must be exceedingly numerous.

In order, therefore, to avoid error as much as possible, I have endeavoured to determine the extreme points, reserving to myself, if it should be desirable, the opportunity of noticing, at a subsequent period, the intermediate facts.

I will therefore state the quantity of the cephalo-spinal liquid, first, as it occurs in the sane; second, as it occurs in the imbecile; and, third, as it occurs in idiots.

The details of the researches which I have made at the Salpetriere, where there are a great number of fools, idiots, and sane women, I am sorry to say, are too numerous to find a place in this paper:—I must be satisfied, therefore, by mentioning the principal results.

In speaking of idiots, I have reference to those who have become so accidentally, not of idiots from birth, in whom there exists some mal-conformation of the nervous system.

The idiots of whom I speak, present a considerable quantity of the fluid. It occupies the surface of the brain, under the form of a thick layer, and distends the cerebral cavities, displacing all the organs which they contain, and particularly the pineal gland, which neither retains its ordinary position, nor performs those functions which I have attributed to it. The aqueduct is also often considerably enlarged. It is in these cases that the cephalo-spinal liquid may be found to amount to six or seven ounces; and the same circumstance obtains in cases of old persons who suffer from mental derangement.

Fools likewise present a large quantity of the liquid; but it does not accumulate upon the surface of the brain. Whatever may be the variety of derangements, whether monomania, hallucination of the mind, furious mania, melancholia, &c., the ventricles are always much distended and enlarged by the cephalo-spinal liquid. In such cases, three ounces of the liquid have been found in the ventricles alone.

The brains of those endowed with reason contain, in a majority of cases, less than an ounce of liquid in their ventricles at the moment of death. From this circumstance it is easy to distinguish the brain of an idiot from that of a sane person.

I was once under the painful necessity of examining the brain of a man of genius who died at an advanced age, but who enjoyed the plenitude of his intellectual faculties. The entire quantity of the cephalo-spinal liquid contained in his brain and spinal canal was scarcely two ounces, and the cerebral cavities did not contain a drachm.

These general results seem to establish the fact, that the development of the mind is in inverse ratio to the quantity of the cephalo-spinal liquid, a circumstance which, to a certain extent, is easy of comprehension, since the quantity of the liquid can not be increased, but at the expense of the mass of the brain, and that in general, superior intelligence is observed in those who have voluminous and well formed brains.

Thus, those persons who have a large head, a high and expanded forehead, and who attach a certain degree of vanity to this conformation, should not be without inquietude respecting the relative proportion of their cephalo-spinal liquid.

I will add in this place, that this liquid should not be too abundant, and that its passage through the cavities of the brain and spine should be perfectly free and uninterrupted. I lately found in the brain of an aged singer, who, after having shone in our theatre, died an idiot in the Salpetriere, an obliteration of the opening by which the liquid entered into the ventricles; and as the brain of this female presented nothing else by which I could explain the disease of her mind, I was led to regard the obliteration of the opening into the ventricles as a cause, or one of the causes of her idiocy."

In concluding, we feel constrained to say, that the paper of which we have stated the leading propositions, is not written in the philosophical spirit, which characterizes many of the preceding memoirs of the same physiologist. It is redundant in conclusions, but barren of facts. Of this he, himself, seems to be aware, and has intimated that want of time and space, had compelled him to withhold many of the *data* from which his deductions were formed. But these facts are precisely what he should have given; especially when he requires us to assent to the existence and active operation of an agent, which we had been accustomed to regard as the mere accidental offspring of disease. We cannot but think, that our author has presumed a little upon the authority of his eminent name, and flattered himself that his *ipse dixit* would be a substitute for facts. And it must be confessed that we are too often disposed to accept of this commutation. Nothing, however, is more unfavorable to the interests of true science; and no exhortation should be more frequently repeated, than that, which arouses us to vigilance in regard to the foundations on which men of genius and popular authority rest their opinions. The power of invention and discovery, is not always associated with a corresponding faculty of judging; and even when these capacities of the mind were, originally, well balanced, the former may at length preponderate. It is the more vivacious of the two; more ambitious; more growing; and may at length attract to itself a part of the supplies that should go to fortify the other. A vivid imagination may not only dazzle the judgment with which it is associated, but mislead the understandings of others; which in medicine is productive of consequences that all should seek to avert. The creations of fancy, presented to the profession as the deductions of a sound philosophical logic, and unconsciously received by the multitude, have done much to retard its healthy progress in every age; and as long as they continue to appear, it will be the duty of reason to distinguish them from the realities of philosophical induction, and cast them out.

